# Commentary: Towards an integrated understanding of general psychopathology—A commentary on Devine et al. (2026)

**DOI:** 10.1002/jcv2.70163

**Published:** 2026-08-21

**Authors:** Ling‐ling Wang, Yi Wang, Raymond C. K. Chan

**Affiliations:** ^1^ School of Psychology Shanghai Normal University Shanghai China; ^2^ Neuropsychology and Applied Cognitive Neuroscience Laboratory State Key Laboratory of Cognitive Science and Mental Health Institute of Psychology Chinese Academy of Sciences Beijing China; ^3^ Department of Psychology University of Chinese Academy of Sciences Beijing China; ^4^ Neuropsychology and Applied Cognitive Neuroscience Laboratory Department of Psychiatry School of Clinical Medicine the University of Hong Kong Hong Kong China; ^5^ Clinical Neuroscience Consortium Queen Mary Hospital the University of Hong Kong Hong Kong China

**Keywords:** developmental, multimodal, neuroimaging, p‐factor, transdiagnostic

Traditional diagnostic systems are greatly challenged by high comorbidity and arbitrary disorder boundaries. These systems showed poor developmental validity, especially for children and adolescents whose manifestations of symptoms shift heavily with age. Research paradigms, including the National Institute of Mental Health's Research Domain Criteria (RDoC) and the Hierarchical Taxonomy of Psychopathology (HiTOP), have revolutionized psychiatric research by moving beyond categorical psychiatric diagnoses. Transdiagnostic dimensional frameworks that quantify shared and distinct mental health risk across diagnostic groups are valuable for understanding the comorbidity (Chan et al., 2025).

The general psychopathology factor (p‐factor) is one of the theoretical and statistical products that are tightly related to this transdiagnostic shift. The p‐factor quantifies a shared latent dimension of diverse psychopathological symptoms (Caspi et al., 2014). The p‐factor is associated with more functional impairment, greater future mental disorder diagnoses, and lower educational outcomes in adolescence and adulthood (Caspi et al., 2014). Extensive work has been conducted to identify the neuroimaging markers of the p‐factor. However, most previous studies were cross‐sectional. In the 2026 Special Issue of JCPP Advances, Devine et al. (2026) provided a timely examination of whether relations between resting‐state functional connectivity (RSFC) networks and the p‐factor across participants would persist over time in preadolescents using a 3‐year longitudinal sample. Moreover, they also tested whether the RSFC networks would be related to the within‐person rate of change in p‐factor (Devine et al., 2026). Results revealed that persistently higher baseline p‐factors correlate with weaker intra‐default mode network (DMN)/dorsal attention network (DAN) connectivity and hyper‐coupling of DMN‐DAN, DMN‐Cingulo‐Opercular network (CON), Ventral Attention Network (VAN)‐CON. The VAN‐DAN, SN‐CON, and DMN‐VAN connectivity was also predicted within‐person p‐factor change over time. Thus, alterations in the communication of nodes within and between the networks involved in self‐referential processing, top–down and bottom–up attention, salience, and cognitive control may relate to general psychopathology trajectories during emerging adolescence.

This commentary is intended to complement the authors' excellent work by highlighting several remaining questions and the following implications concerning the search for neurobiological correlates of general psychopathology. First, it remains unclear about the full range of psychopathological dimensions that are required to generate a p‐factor measurement. Most existing studies focus heavily on internalizing and externalizing problems such as anxiety, depression, and conduct difficulties, while rarely including measures tapping into the psychotic‐spectrum. Notably, subclinical manifestations of the psychotic spectrum, such as social anhedonia and decreased pleasure experience from social interactions, are closely related to negative functional outcomes and a higher risk of developing various psychiatric disorders. Psychotic disorders also have high comorbidity with disorders like bipolar disorder, obsessive‐compulsive disorder etc. Thus, incorporating these symptom domains may therefore enrich the comprehensive coverage of general psychopathology. This common omission is possibly attributable to the widespread use of general population samples in prior research, in which the prevalence of psychotic symptoms and disorders may be too low for reliable statistical modelling, particularly among children and adolescents (Michelini et al., 2019). For the ABCD cohort, psychotic symptom items from the Child Behaviour Checklist are frequently excluded during exploratory factor analysis due to low prevalence, which prevents their reliable integration into p‐factor and other psychopathology dimensions. Accordingly, this highlights a critical need for future studies to recruit clinical samples and psychopathology‐enriched samples to better capture the psychotic spectrum. In addition, questions remain unclear about the different p‐factors extracted from different symptom clusters. A p‐factor estimated from a broader set, including internalizing, externalizing, and psychotic‐spectrum symptoms, may be different from a p‐factor estimated solely from internalizing and externalizing problems. It is possible that the range of measured symptoms may shape the p‐factor construct. Consequently, neural correlates of a specific p‐factor may be limited to several symptoms and may not generalize to the broader transdiagnostic construct. This reinforces our argument that future research should integrate a more comprehensive measurement of general psychopathology.

Second, much of the existing research on the p‐factor is built upon youth samples recruited from Western cultural contexts. Cultural norms may shape how emotion, behaviour, and psychotic experiences are subjectively perceived, reported, and expressed. It is important to consider that cultural norms may in turn influence the correlational structure of symptom items that were used to derive the p‐factor. Future research is encouraged to utilize cross‐cultural designs for further validation of the p‐factor. Several Asian economies have launched large‐scale national brain initiatives that enable cross‐cultural validation of the p‐factor, such as the Korea Brain Initiative (Jeong et al., 2016). The ongoing China Brain Project also offers a unique and large‐scale research platform equipped with multi‐modal neuroimaging and long‐term developmental follow‐up data of Chinese child and adolescent cohorts (Poo et al., 2016). These national brain cohorts may provide valuable platforms to examine whether the general p‐factor exhibits consistent structure across populations.

Third, developmental research on the p‐factor could extend beyond the transition from childhood to early adolescence to also consider the developmental shift from adolescence into adulthood (Choate et al., 2023), a period encompassing the peak onset of multiple psychiatric disorders, including schizophrenia and major depressive disorder. By targeting this specific stage, we can gain more insights regarding how the p‐factor and its fluctuations may be linked with the onset of mental disorders. More importantly, if the p‐factor reflects a transdiagnostic latent dimension of psychopathology, it may also be observable across other life stages such as middle and older adulthood. Longitudinal research adopting a lifespan perspective would therefore be valuable for advancing understanding of general psychopathology. A key methodological challenge is establishing a generalized evaluation tool suitable for lifespan‐wide population assessment.

In parallel, neuroimaging studies exploring neural correlates of the p‐factor may benefit from a multimodal approach (Plitman et al., 2020). Resting‐state functional MRI captures spontaneous neural activity under unconstrained conditions. Despite its accessibility and importance, it may fail to reflect context‐dependent cognitive and emotional deficits linked to psychopathological vulnerability. Incorporating other imaging modalities, such as task‐based functional MRI, structural MRI, diffusion tensor imaging, and magnetic resonance spectroscopy, can provide multi‐level data spanning task‐related brain activity, brain structure, and neurochemical concentrations. Integrating these diverse neural measures may help systematically unpack the neurobiological mechanisms underlying the p‐factor. Importantly, although accumulating evidence has established the neural correlates of the p‐factor, the interplay between brain maturation and the emergence and changes of the p‐factor remains poorly understood. It is still unclear how brain alterations across childhood, adolescence, and adulthood shape the developmental trajectory of the p‐factor. Relatedly, whether there exists some distinct neural characteristic that can constrain or facilitate the expression of general psychopathology is also unknown. This leaves considerable room for future longitudinal multimodal neuroimaging research to clarify.

Moreover, the conceptual interpretation of the p‐factor remains a debate. The p‐factor is conceptually parallel to Spearman's well‐documented g‐factor. The g‐factor is suggested to be a shared cognitive resource that supports performance across diverse cognitive tasks. Similarly, some p‐factor research adopted a common cause perspective, which frames the p‐factor as a stable liability or deficit that precedes the specific occurrence of disorder. However, there were also different views regarding the nature of the p‐factor. For example, the dynamic mutualism theory offers an alternative point of view to interpret the p‐factor. This framework posits that the statistical correlation observed across the diverse symptoms may arise from reciprocal, mutually reinforcing interactions between these symptoms, rather than stemming solely from one unified underlying predisposition (Van Bork et al., 2017). The high covariation between different symptoms may be the result of a high common factor. However, it can also be explained using mutualism so that having more severe internalizing problems brings about more sleep difficulties, and more sleep difficulties further worsen the internalizing problems.

In the 2026 Special Issue of JCPP Advances, Peng and Watts (2026) directly tested the common cause and the dynamic mutualism theories in explaining the associations between aspects of ADHD and the neurodevelopmental and externalizing spectra across four waves of the ABCD data (Peng & Watts, 2026). The authors found more support for the common cause model compared with dynamic mutualism. However, another study has found preliminary support for the dynamic mutualism processes in the development of co‐occurring psychopathology (Choate et al., 2023). Such conflicting empirical findings highlight that existing evidence remains inconclusive. It could be that atypical cognitive processes or brain function are markers of developing a psychiatric disorder. Alternatively, they can also be the descending results of accumulating disease burden or distress. Future research adopting multi‐wave longitudinal designs with both neuroimaging and symptom assessments may be vital to test these competing predictions. In addition to p‐factor, future research could also report findings from different symptom domains such as internalizing, externalizing, and psychotic symptoms.

Taken together, the transdiagnostic framework has advanced psychiatric research by overcoming the limitations of traditional categorical diagnostic systems. Devine et al. (2026) delineated the prospective associations of RSFC and p‐factor trajectories in adolescents. Their results indicated that RSFC in cognitive and attentional control networks may be potential markers and intervention targets of general psychopathology risk. Complementing their important work, we highlighted that future studies should integrate comprehensive transdiagnostic symptom assessments. Cross‐cultural and lifespan longitudinal studies are core to understanding the role of p‐factor in shaping the psychopathology. More importantly, we emphasized that competing explanations of p‐factor, such as a latent liability and a result of dynamic mutualistic interaction, should be empirically tested. In turn, these can refine the theoretical interpretation of general psychopathology and promote the transdiagnostic prevention and intervention of mental disorders.

## AUTHOR CONTRIBUTIONS

**Ling‐ling Wang:** Writing—original draft preparation (equal). **Yi Wang:** Writing—original draft preparation (equal). **Raymond C. K. Chan:** Conceptualization; writing—review and editing.

## CONFLICT OF INTEREST STATEMENT

The authors declare no conflicts of interest.

## ETHICAL CONSIDERATIONS

Ethical approval is not applicable for this commentary article.

## Data Availability

Data sharing not applicable to this article as no datasets were generated or analysed for this commentary article.
